# The impact of non-medical cannabis legalization and other exposures on retention in longitudinal cannabis research: a survival analysis of a prospective study of Canadian medical cannabis patients

**DOI:** 10.1186/s42238-021-00089-7

**Published:** 2021-07-28

**Authors:** Philippe Lucas, Susan Boyd, M.-J. Milloy, Zach Walsh

**Affiliations:** 1 Social Dimensions of Health, University of Victoria, 3800 Finnerty Rd, Victoria, B.C V8P 5C2 Canada; 2 1100 Maughan Rd, Nanaimo, BC V9X1J2 Canada; 3 Canadian Institute for Substance Use Research, 2300 McKenzie Ave, Victoria, BC V8N 5M8 Canada; 4 Faculty of Human and Social Development, School of Public Health and Social Policy, University of Victoria, 3800 Finnerty Rd, Victoria, B.C V8P 5C2 Canada; 5 Faculty of Medicine, University of British Columbia, St. Paul’s Hospital, Burrard Street, Vancouver, B.C 806-1081 Canada; 6 British Columbia Centre On Substance Use, 400-1045 Howe St, Vancouver, B.C V6Z 2A9 Canada; 7 Department of Psychology, University of British Columbia, 3333 University Way, OkanaganKelowna, B.C V1V 1V7 Canada; 8 Centre for the Advancement of Psychological Science and Law, University of British Columbia, 3333 University Way, OkanaganKelowna, BC V1V 1V7 Canada

**Keywords:** Cannabis, Marijuana, Retention, Legalization, Survival analysis, Drop out, Clinical trial

## Abstract

**Background:**

Despite repeated calls by medical associations to gather evidence on the harms and benefits of cannabis, there are ongoing methodological challenges to conducting observational and clinical studies on cannabis, including a high rate of patients that are lost to follow-up (LTFU). This study explores factors potentially associated with retention in a large prospective study of Canadian medical cannabis patients, with the goal of reducing the probability that patients will be lost to follow-up in future cannabis research.

**Methods:**

The Tilray Observational Patient Study (TOPS) was a multi-site, prospective study assessing the impact of medical cannabis over 6 months in a broad population of authorized Canadian cannabis patients. The study took place from 2016 to 19, and we conducted a series of exploratory analyses including a Kaplan–Meier survival analysis and logistic regressions to assess the potential association between study retention and variables including patient characteristics, cannabis and prescription drug use, quality of life, and the legalization of non-medical cannabis.

**Results:**

Overall, 1011 participants were included in this analysis, contributing 287 patient-years of data. Retention was 728 (72%) at 3 months, and 419 (41.4%) at 6 months. Our analyses found significantly lower adjusted odds of retention following legalization (AOR 0.28, 95% CI 0.18–0.41), and in patients that used prescription opioids at baseline (AOR 0.62, 95% CI 0.46–0.85), while increased odds of retention were found in patients with a higher baseline psychological score (AOR 1.43, 95% CI 1.08–1.90) or that used anti-seizure medications at baseline (AOR 1.91, 95% CI 1.30–2.81).

**Discussion:**

TOPS provided a unique opportunity to examine patient characteristics and other variables that may be associated with retention in prospective medical cannabis studies. Our findings highlight some of the challenges of conducting medical cannabis research at a time when patients have a multitude of cannabis access options, including legal adult dispensaries and a robust illicit market. High LTFU rates can impact the validity of studies, and potentially lead to misestimations of the harms and benefits of medical cannabis use. Despite being a multi-site prospective study, this was a convenience sample, thereby limiting the generalizability of these findings. Additionally, data regarding the use of cannabis was self-reported by patients, so is subject to potential recall bias.

**Conclusion:**

We found evidence that external policy changes that affect access to cannabis such as the legalization of non-medical adult use and patient characteristics associated with patient physical/psychological capacity can impact retention in prospective medical cannabis studies. Evidence-based strategies to reduce study burden on participants, such as minimizing in-person visits by providing digitized internet-based surveys and phone or telemedicine follow-up options as well as ensuring adequate participant compensation could improve retention. Additionally, policy-related changes aimed at improving access to medical cannabis, including increased cost-coverage and community-based distribution, could encourage patients to remain in the federal medical cannabis program and thereby reduce LTFU in associated studies.

**Supplementary Information:**

The online version contains supplementary material available at 10.1186/s42238-021-00089-7.

## Background

In 2001, Canada became one of the first nations to regulate medical cannabis at a federal level, and in 2018 it was the second country to legalize the non-medical use of cannabis by adults (Fischer et al., 2020; Lucas et al., 2019). Despite consistent calls by the Canadian Medical Association and American Medical Association citing the need for high-quality studies to identify the risks and benefits of cannabis in both medical and non-medical applications (Cannabis | CMA Health Topics, 2020; Harris, 2019), there are many historical and ongoing challenges to conducting such research. As a result, many systematic reviews examining the available evidence in a number of primary therapeutic applications for cannabis have cited both a lack of and need for well-designed longitudinal observational and controlled studies (Bonaccorso et al. 2019; Hoch et al. 2019; Kosiba et al., 2019; Okusanya et al. 2020).

There are a number of challenges to conducting high-quality cannabis research in Canada and in other jurisdictions. These include social stigma resulting from the long-standing international prohibition on cannabis possession and use (Belle-Isle et al. 2014; Bottorff et al. 2013; Lucas, 2009), a lack of funding and regulatory obstacles and associated delays National Academies of Sciences, Engineering, and Medicine; Health and Medicine Division; Board onPopulation Health and Public Health Practice; Committee on the Health Effects of Marijuanav, 2017; Geary 2019), and methodological difficulties, such as the inability to blind THC-based products in controlled studies due to its potential for impairment (Russo 2016; National Academies of Sciences, Engineering, and Medicine; Health and Medicine Division; Board on Population Health and Public Health Practice; Committee on the Health Effects of Marijuana, 2017). Additionally, legalizing the adult, non-medical use of cannabis in Canada in October 2018 has significantly changed how medical and non-medical adult users access cannabis, and this may have subsequent impacts on recruitment, adherence, and retention in prospective medical cannabis studies in Canada.

The legalization of the adult non-medical use of cannabis in Canada is in its nascency, and the federal, provincial, and municipal policies governing access continue to evolve. However, some data suggests that one of the impacts of legalization has been a decline in participation in the federal medical cannabis program. Recent statistics from Health Canada show that the number of authorized patients peaked in September 2019 at 369,614 (although legalization took place in October 2018, medical cannabis recommendations by health care practitioners are valid for a maximum of 12 months, so a decline in patient renewals would be staggered accordingly), declining steadily to 303,221 in June 2020, the latest month for which data was available (Health Canada 2020). This steady migration away from the medical cannabis program may be due to a number of factors. Over the years, many barriers have been identified in accessing medical cannabis in Canada, including stigma, lack of support from the medical community, limited product selection, and high costs (Belle-Isle and Hathaway, 2007; Bottorff et al. 2013; Capler et al. 2017; Lucas, 2009). By contrast, both the illicit market as well as the legal, non-medical cannabis dispensaries offer many advantages not currently available in the mail-order only federal medical cannabis program, including the opportunity for in-person, community-based interactions, a large selection of products from hundreds of different producers, and highly competitive pricing. Furthermore, a few studies examining the impact of legalization on patient access to medical cannabis report that product shortages in the medical system immediately following legalization led patients to purchase cannabis from non-medical sources, including the illicit market (McTaggart-Cowan et al. 2020; Hawley 2020). This research and data suggest a need to examine if the increase in access options following legalization could also be affecting retention in prospective medical cannabis studies.

Therefore, we conducted an exploratory analysis of a large prospective study of medical cannabis patients to examine the potential relationship between study retention and respondent characteristics, psychosocial factors, cannabis use, and the use of prescription drugs. Additionally, we examined whether the legalization of non-medical adult cannabis use impacted the rates of patients lost to follow-up (LTFU). Our objectives were to identify factors associated with retention, and to recommend clinical strategies and policy options that might reduce LTFU in future longitudinal medical cannabis studies. Moreover, our findings may prove useful in contextualizing existing findings on the outcomes of cannabis use in other longitudinal studies.

## Methods

The Tilray Observational Patient Study (TOPS) was a national, multi-site, prospective medical cannabis study that took place at 21 medical clinics in five Canadian provinces, with the goal of gathering detailed information on patient characteristics and examining the impact of medical cannabis use on quality of life and prescription drug use over 6 months. TOPS used a pre-test/post-test repeated measures design, with data gathering at baseline, 1 month, 3 months, and 6 months. The study was reviewed and approved by Advarra (formerly Institutional Review Board Services) on 22 January 2016, the University of Victoria on 7 April 2016, and the Alberta Health Research Ethics Board of Alberta 3 October 2016, and sponsored by Tilray, a licensed medical cannabis production and research company based in British Columbia, Canada.

Physicians identified, recruited, and screened patients in-clinic during regularly scheduled appointment, guided by ethics-approved Health Care Provider Talking Points provided by the study sponsor (Additional file 1). Participants were federally authorized, English speaking medical cannabis patients 18 years old and over with the capacity to consent for themselves who received a new cannabis recommendation from a participating physician, and subsequently registered with Tilray to obtain their medical cannabis products via mail. As compensation for their time, participants received a $25 credit towards their medical cannabis costs after completing each set of surveys. Clinics and participating physicians were identified and trained in the administration of the study by the principal investigator and colleagues, and data was gathered digitally via REDCap, a secure electronic data capture system (Harris et al. 2019). Study analyses included 1011 participants who enrolled in TOPS before 16 July 2018 to ensure all those included had the opportunity to be in the study at least 6 months and therefore could have completed all study visits.

### Measures

TOPS was composed of a combination of validated and novel instruments made up of multiple-choice questions, rating and rankings, visual assessment scales (VAS) and Likert scales, as well as matrix and dropdown questions. Many questions also included an “other” option that then provided a text box for short textual responses. The primary outcome of interest for this analysis was retention in the study at 6 months, and the explanatory variables we hypothesized may be related to retention included primary patient demographics, baseline quality of life scores, and patterns of medical cannabis and prescription drug use, as well as whether patients participated in the study prior to or after the legalization of adults non-medical use of cannabis in Canada on 17 October 2018.

Demographic data (such as age, gender, marital status, education level, employment status, and province of residence) and primary condition were self-reported and gathered via multiple choice questions informed by past longitudinal and cross-sectional surveys (Lucas et al. 2019; Lucas and Walsh, 2017; Walsh et al. 2013). The study included three additional instruments: the World Health Organization Quality of Life Short Form, the Cannabis Use Survey, and the Prescription Drug Questionnaire.

The World Health Organization Quality of Life Short Form (WHOQOL-Bref) (WHOQOL Group, 1998) is a validated 26-item questionnaire derived from data collected using the WHOQOL-100. It produces scores for four domains related to quality of life: physical health, psychological, social relationships, and environment. (Saxena et al. 2001). The Cannabis Use Survey (CUS) is a 17-question self-administered patient questionnaire designed to gather cross-sectional and/or prospective information on medical cannabis patient primary conditions, symptoms, and patterns of medical cannabis use such as amounts used, preferred methods of use, and cannabis type preferences (e.g., THC vs. CBD). The Prescription Drug Questionnaire (PDQ) was designed to produce an accurate inventory of current health care provider authorized prescription drug use by a patient, and is completed by physicians and/or medical clinic staff in cooperation with the patient in order to limit the potential for recall bias. It gathers detailed information on daily and non-daily prescription drug use in milligrams per dose, and doses per day or week (where applicable), and has an auto-fill function connected to the National Drug Data File (NDDF), a US-based national prescription drug database, to ensure that consistent generic prescription drug names are used across participants and medical clinics in order to facilitate analysis.

This battery was administered at four different time points: at baseline after a patient has received a medical cannabis recommendation from the participating physician, and then at 1 month (M1), 3 months (M3), and 6 months (M6). If a patient missed a study visit, they were nonetheless contacted and given the opportunity to continue with the study at the next time point.

### Data analysis

Summary statistics were calculated for the following patient characteristics: age, gender, education level and marital status, as well as for primary condition, and past/present cannabis usage (naïve vs. non-naïve; method of use; frequency of use). Cannabis-naïve patients were defined as those who had used cannabis four times or fewer in the previous 12 months.

In order to assess quality of life, the four domains of the WHOQOL-Bref were tabulated at each study visit, and mixed-effects linear regression was used to model the time trend of the four domains over the 6-month period for all patients, as well as by different levels of demographic variables and other baseline patient characteristics.

The analysis of prescription drug use data included descriptive summaries of the number and percentage of patients who used each medication stratified by baseline usage of the particular medication, as well as by patient characteristics. To assess the dosage and usage frequency data among those who used the medication at baseline, milligrams (mg) per dose was first converted to milligrams per day by multiplying milligrams per dose with the frequency per day. The reported dosage for each drug was then divided by its defined daily dose (DDD) to facilitate a summary of dosage data across patients (WHO Collaboration Center for Drug Statistics Methodology 2019), and then the most prevalent drugs were grouped into five primary drug classes for further analysis: opioid and non-opioid pain medications, benzodiazepines, antidepressants, and anti-seizure drugs.

As the survival analysis focused on two time points (M3 and M6), the Kaplan–Meier method and log-rank test were used, with the primary outcome of interest being retention at M6. If a patient did not complete both M3 and M6, the patient was considered to be dropped out at M3. If a patient completed M3 visit but not M6, the patient was considered to be dropped out at M6. The completion rate of M6 was initially summarized by patient characteristics, change in QOL, and change in drug usage. In order to facilitate the logistic regression and Kaplan–Meier analysis, we re-defined some measures such as QOL as binary outcomes using the median split of the 1011 patients included in this analysis. The Kaplan–Meier estimator then reported the probability of remaining in the study at M3 and M6, and we used the log-rank test for between group comparisons. Groups with less than five patients were not assessed.

We then proceeded with a univariate logistic regression analysis in order to better understand the association between the variables assessed in the Kaplan–Meier estimator and retention at M6, with the addition of a binary variable to assess potential impact of legalization by comparing retention between patients enrolled prior to 17 April 2018 (so that M6 would be before legalization) and those enrolled between 17 April to 15 July 2018 (so M6 would be post-legalization). This was followed by a multivariate analysis that included all significant variables from the univariate analysis, using a chi-square test for homogeneity.

All analyses were conducted in SAS 9.4 (SAS Institute, Cary, NC, USA) and/or R 3.6.3 (R Core Team). All statistical tests were two-sided, with significance levels of 0.05 unless otherwise indicated.

## Results

Overall, 1011 were included in this analysis and contributed 287 patient-years of observation. Retention was 728 (72%) at 3 months, and 419 (41.4%) at 6 months. Tables 1 and 2 provide an overview of the primary baseline characteristics of the 1011 patients included in this analysis, along with the associated percentage that remained in the study at M6, and the results of the Kaplan–Meier estimator probability of remaining in the study at M3 and M6. Most participants were female (578, 57%) and 560 (55%) having at least a college degree. The median age was 51.0 (IQR 38–61) at baseline, and most were married or equivalent (561, 56%). The top 5 primary conditions reported by participants were chronic pain (703, 70%), anxiety disorders (98, 10%), arthritis (63, 6%), insomnia (48; 5%), and headache (23, 2%). Therefore, pain, mental health issues, and insomnia accounted for approximately 94% of all participant primary conditions. These participant characteristics are largely consistent with previous Canadian and international studies of medical cannabis patients (Hazekamp et al. 2013; Baron et al. 2018; Boehnke et al. 2019; Lintzeris et al. 2020). No statistically significant associations were found between patient characteristics such as gender, education, marital status, age, previous cannabis experience, and primary condition and the probability of remaining in the study.

**Table 1:** Percent completing M6 and probability of retention at M3 and M6 by patient baseline characteristics and primary condition in 1011 participants

**Patient baseline characteristics**	**Completed M6 (%)**	**Kaplan–Meier estimator**		
		**Probability of remaining in study**		
		**M3**	**M6**	***p***
**Gender**				0.382
Male	175/432 (40.5)	0.70	0.41	
Female	244/578 (42.2)	0.74	0.42	
**Education**				0.299
High school or lower	178/451 (39.5)	0.71	0.39	
College or higher	241/560 (43.0)	0.73	0.43	
**Marital status**				0.272
Single/divorced/widowed/separated	175/450 (38.9)	0.72	0.39	
Married/living as married	244/561 (43.5)	0.72	0.43	
**Age**				0.279
< 25	5/18 (27.8)	0.78	0.28	
25–39	102/264 (38.6)	0.69	0.39	
40–55	141/345 (40.9)	0.70	0.41	
> 55	171/384 (44.5)	0.75	0.45	
**Age**				0.107
< 55	238/602 (39.5)	0.70	0.40	
≥ 55	181/409 (44.3)	0.74	0.44	
**Used cannabis 5 or more times in the last 12 months**				0.459
No	245/580 (42.2)	0.73	0.42	
Yes	168/415 (40.5)	0.70	0.40	
**Primary medical condition you currently treat with medical cannabis**				0.913
Anxiety disorder	37/98 (37.8)	0.73	0.38	
Arthritis	27/63 (42.9)	0.75	0.43	
Cancer/leukemia	8/15 (53.3)	0.73	0.53	
Chronic pain	298/703 (42.4)	0.72	0.42	
Crohn’s disease	1/6 (16.7)	0.67	0.17	
Epilepsy	3/5 (60.0)	0.80	0.60	
Gastrointestinal disorder	3/6 (50.0)	0.83	0.50	
Headache	8/23 (34.8)	0.78	0.35	
Insomnia	20/48 (41.7)	0.65	0.42	
Movement disorder	2/8 (25.0)	0.63	0.25	
PTSD	2/8 (25.0)	0.63	0.25	
Other	8/18 (44.4)	0.78	0.44	
**Primary medical condition you currently treat with medical cannabis**				0.862
Pain	333/789 (42.2)	0.72	0.42	
Mental health issues	40/109 (36.7)	0.73	0.37	
Insomnia	20/48 (41.7)	0.65	0.42	
Other	26/64 (40.6)	0.77	0.41	

*M3*  Month 3 timepoint, *M6*  Month 6 timepoint

**Table 2:** Percent completing M6 and probability of retention at M3 and M6 by baseline prescription drug use and quality of life (QOL) in 1011 participants

**Baseline prescription drug use and QOL**	**Completed M6 (%)**	**Kaplan–Meier estimator**		
		**Probability of remaining in study**		
		**M3**	**M6**	***P***
**Use of opioid**				0.004
No	306/697 (43.9)	0.74	0.44	
Yes	102/290 (35.2)	0.66	0.35	
**Opioid – defined daily dose (DDD)**				0.321
< 0.57	44/108 (40.7)	0.64	0.41	
≥ 0.57	34/108 (31.5)	0.66	0.31	
**Use of non-opioid pain medications**				0.721
No	314/770 (40.8)	0.73	0.41	
Yes	94/215 (43.7)	0.70	0.44	
**Non-opioid pain medications—defined daily dose (DDD)**				0.245
< 0.43	37/76 (48.7)	0.72	0.49	
≥ 0.43	36/89 (40.4)	0.64	0.40	
**Use of benzodiazepine**				0.151
No	375/918 (40.8)	0.71	0.41	
Yes	32/67 (47.8)	0.82	0.48	
**Benzodiazepine–defined daily dose (DDD)**				0.151
< 0.38	13/29 (44.8)	0.71	0.41	
≥ 0.38	15/27 (55.6)	0.82	0.48	
**Use of antidepressant**				0.011
No	324/818 (39.6)	0.71	0.40	
Yes	83/166 (50.0)	0.78	0.50	
**Antidepressant—defined daily dose (DDD)**				0.342
< 1	14/35 (40.0)	0.74	0.40	
≥ 1	45/90 (50.0)	0.78	0.50	
**Use of anti-seizure**				0.003
No	326/827 (39.4)	0.71	0.39	
Yes	83/159 (52.2)	0.79	0.52	
**Anti-seizure—defined daily dose (DDD)**				0.542
< 0.5	24/46 (52.2)	0.80	0.52	
≥ 0.5	34/72 (47.2)	0.75	0.47	
**WHOQOL—physical health (baseline)**				0.671
< 36	218/526 (41.4)	0.74	0.41	
≥ 36	199/481 (41.4)	0.70	0.41	
**WHOQOL—psychological (baseline)**				0.042
< 54	166/442 (37.6)	0.71	0.38	
≥ 54	251/565 (44.4)	0.73	0.44	
**WHOQOL—social relationships (baseline)**				0.168
< 58	147/384 (38.3)	0.71	0.38	
≥ 58	270/623 (43.3)	0.72	0.43	
**WHOQOL—environment (baseline)**				0.166
< 66	222/556 (39.9)	0.70	0.40	
≥ 66	195/451 (43.2)	0.75	0.43	

*M3* = month 3 timepoint, *M6* = month 6 timepoint

Table 2 highlights the rate of study completion and probability of retention at M3 and M6 by baseline prescription drug use and quality of life. Overall, 283 participants were LTFU at M3, and a further 309 were LTFU at M6. The five most commonly used prescription drug classes were opioids, with 290 (29.4%) reporting baseline opioid use, followed by non-opioid pain medications (215, 21.8%), antidepressants (166, 16.9%), anti-seizure drugs (159, 16.1%), and benzodiazepines (67, 6.8%).

Baseline opioid use was associated with lower rates of completion, with 44% of non-opioid users completing M6 (*n* = 306) compared to 35% of opioid users (*n* = 102). This was also reflected in the lower probability of people reporting opioid use remaining in the study at M3 and M6 (66% at M3 and 35% at M6 for opioid users compared to 74% and 44% for non-opioid users; *p* = 0.004). However, the use of antidepressants and of anti-seizure drugs were both associated with greater percentage of M6 completion, and associated increases in probability of retention at M3 and M6. Probability of retention at M3 and M6 for those using antidepressants was 78% and 50%, respectively, compared to 71% at M3 and 40% at M6 for those not using antidepressants (*p* = 0.011). Those using anti-seizure medications saw similar outcomes: 79% (M3) and 52% (M6) compared to 71% and 39% for non-users (*p* = 0.003).

For quality of life, median baseline scores were used to create a binary comparison, and only a higher baseline score for the psychological measure of WHOQOL-Bref was associated with increased probability of remaining in the study. Those scoring 54 or better had a 73% probability of remaining in the study at M3, and 44% at M6, compared to 71% at M3 and 38% at M6 for those who scored below 54 (*p* = 0.042).

Table 3 highlights the rate of study completion and probability of retention at M3 and M6 by baseline medical cannabis use. Median weekly cannabis use was 5.0 g (IQR 2.0–7.5). In regard to preferred types of cannabis products, the most cited was high CBD (461, 46.8%), and more patients cited “no preference” (286, 29%) than those who identified a preference for THC (239, 24.2%). For primary method of use, oral ingestion was cited by 546 (54.7%) while inhaled methods (e.g., vaporizers, joints, pipes, and bongs) accounted for 44.6%. Additionally, most participants reported using at least some extract (orally ingested) products (619, 61.9%).

**Table 3:** Percent completing M6 and probability of retention at M3 and M6 by baseline medical cannabis use in 1011 participants

**Baseline cannabis use**	**Completed M6 (%)**	**Kaplan–Meier estimator**		
		**Probability of remaining in study**		
		**M3**	M6	***P***
**Cannabis use per week (g)**				0.567
< 5	211/495 (42.6)	0.74	0.43	
≥ 5	208/505 (41.2)	0.72	0.41	
**Frequency of cannabis use per week**				0.587
< 14	186/428 (43.5)	0.72	0.43	
≥ 14	233/571 (40.8)	0.73	0.41	
**Currently using Tilray extract products**				0.751
No	165/381 (43.3)	0.71	0.43	
Yes	254/619 (41.0)	0.74	0.41	
**Preferred type of cannabis**				0.044
THC	105/239 (43.9)	0.72	0.44	
High CBD	174/461 (37.7)	0.73	0.38	
No preference	139/286 (48.6)	0.74	0.49	
**Primary method of use**				< 0.001
Vaporizer—cannabis flower	99/167 (59.3)	0.80	0.59	
Vaporizer/nail—cannabis extracts	2/10 (20.0)	0.50	0.20	
Joint	73/194 (37.6)	0.71	0.38	
Oral	209/546 (38.3)	0.72	0.38	
Pipe	11/33 (33.3)	0.58	0.33	
Waterpipe/bong	18/35 (51.4)	0.80	0.51	
Topical	5/11 (45.5)	0.82	0.45	
**Primary method of use**				0.027
Inhaled	203/439 (46.2)	0.74	0.46	
Orally ingested	209/546 (38.3)	0.72	0.38	

*M3* = month 3 timepoint, *M6* = month 6 timepoint

Citing no preference for either THC or CBD significantly increased the probability of retention at M6 (49% for no preference vs. 44% for THC and 38% for CBD, *p* = 0.044), as did inhalation vs. oral ingestion (46% for inhalation vs. 38% for oral ingestion, *p* = 0.027). However, this analysis found no association between the amount of cannabis used per week, frequency of use per week, and reporting use of extract products, and the probability of retention at M3 or M6.

Table 4 presents the results of the univariate logistic regression as the unadjusted odds of completing M6 by baseline patient characteristics and other variables of interest, including the legalization of non-medical adult use of cannabis in Canada. Overall, primary patient characteristics such as gender, education, marital status, age, previous experience with cannabis, and primary condition were not found to be associated with retention. However, baseline prescription drug use, quality of life, aspects of cannabis use, and cannabis legalization were associated with significant impacts on the odds of completing M6 (Table 4).

**Table 4:** Unadjusted odds of completing M6 in 1011 participants by baseline patient characteristics and other variables

Patient characteristics	Univariate analysis		
	**OR**^**a**^	**95% CI**^**b**^	***P***
**Gender**			
Female	—	—	
Male	0.93	0.72, 1.20	0.587
**Education**			
College or lower	—	—	
High school or lower	0.86	0.67, 1.11	0.252
**Marital status**			
Married/living as married	—	—	
Single/divorced/widowed/separated	0.83	0.64, 1.06	0.140
**Age**			
< 55	—	—	
≥ 55	1.21	0.94, 1.57	0.135
**Used cannabis for any reason 5 or more times in the last 12 months**			
No	—	—	
Yes	0.93	0.72, 1.20	0.579
**Primary illness or medical condition you currently treat with medical cannabis**			
Pain	—	—	
Mental health issues	0.79	0.52, 1.20	0.275
Insomnia	0.98	0.54, 1.77	0.942
Other	0.94	0.56, 1.57	0.805
**Use of opioid**			
N	—	—	
Y	0.69	0.52, 0.92	0.011
**Use of non-opioid pain medications**			
N	—	—	
Y	1.13	0.83, 1.53	0.439
**Use of benzodiazepine**			
N	—	—	
Y	1.32	0.81, 2.18	0.269
**Use of antidepressant**			
N	—	—	
Y	1.52	1.09, 2.13	0.014
**Use of anti-seizure**			
N	—	—	
Y	1.68	1.19, 2.36	0.003
**Opioid—dose per day (DDD)**			
> 0, < 0.57	—	—	
≥ 0.57	0.67	0.38, 1.17	0.157
**Non-opioid pain medications—dose per day (DDD)**			
> 0, < 0.43	—	—	
≥ 0.43	0.72	0.39, 1.33	0.289
**Benzodiazepine—dose per day (DDD)**			
> 0, < 0.38	—	—	
≥ 0.38	1.54	0.54, 4.42	0.423
**Antidepressant—dose per day (DDD)**			
> 0, < 1	—	—	
≥ 1	1.50	0.68, 3.31	0.316
**Anti-seizure—dose per day (DDD)**			
> 0, < 0.5	—	—	
≥ 0.5	0.82	0.39, 1.72	0.60
**WHOQOL—physical health (baseline)**			
< 36	—	—	
≥ 36	1	0.78, 1.28	0.981
**WHOQOL—psychological (baseline)**			
< 54	—	—	
≥ 54	1.33	1.03, 1.71	0.028
**WHOQOL—social relationships (baseline)**			
< 58	—	—	
≥ 58	1.23	0.95, 1.60	0.114
**WHOQOL—environment (baseline)**			
< 66	—	—	
≥ 66	1.15	0.89, 1.47	0.289
**Cannabis use per week (g)**			
< 5	—	—	
≥ 5	1.06	0.83, 1.36	0.645
**Frequency of cannabis use per week**			
< 14	—	—	
≥ 14	1.11	0.87, 1.44	0.401
**Currently using Tilray extract products**			
N	—	—	
Y	0.91	0.70, 1.18	0.479
**Preferred type of cannabis**			
CBD	—	—	
THC	1.29	0.94, 1.78	0.113
No preference	1.56	1.16, 2.10	0.004
**Primary method of use**			
Inhaled	—	—	
Orally ingested	0.72	0.56, 0.93	0.012
**Enrollment period**			
Enrolled prior to 17 April 2018 (M6 would be pre-legalization)	—	—	
Enrolled between 17 April and 15 July 2018 (M6 would be post legalization)	0.32	0.22, 0.46	< 0.001

^**a**^*OR*  Odds ratio, ^b^*CI* Confidence interval

Using opioids was associated with lower unadjusted odds of completing the study (*p* = 0.011), and this outcome appeared to be independent of the daily dose used by participants. Conversely, patients using antidepressants had greater odds of retention (*p* = 0.014), as did those using anti-seizure medication (*p* = 0.003); however, as with opioids, these outcomes were not associated with specific defined daily doses. In regard to baseline quality of life as measured by WHOQOL-Bref, a score of 54 or more in the psychological domain was associated with greater odds of retention compared to those scoring below 54 (*p* = 0.028). None of the other three domains assessed—physical health, social relationships, or environment—appeared to be associated with retention.

Specific to cannabis use, citing no preference for either THC or CBD was associated with greater odds of retention compared to citing a preference for CBD, while a preference for THC was not associated with retention (*p* = 0.113). Moreover, using cannabis via oral ingestion was found to result in lower odds of completing M6 compared to those who inhale cannabis as their primary method of use (*p* = 0.012).

Finally, cannabis legalization was found to have a significant impact on retention at M6. Participants who enrolled in the study in the period that would have resulted in M6 being after legalization had significantly lower odds of completing the study than those who enrolled prior to that period (*p* < 0.001).

Table 5 presents the results of a multivariate model that included all variables found to be significant in the univariate analyses. The primary factor impacting retention was legalization (AOR = 0.28, 95% CI 0.18–0.41). Additionally, the use of opioids continued to be significantly associated with reductions in the adjusted odds of retention at M6 (AOR = 0.62, 95% CI 0.46–0.85). Moreover, the use of anti-seizure medications continued to be associated with significantly greater adjusted odds of retention at M6 (AOR 1.91, 95% CI 1.08–1.90), as was WHOQOL-Bref psychological scores of ≥ 54 (AOR 1.43, 95% CI 1.08–1.90), with both associations actually increasing in the multivariate model. However, this analysis also found that the use of antidepressants was no longer associated with retention (*p* = 0.061), nor was using a specific primary method of use, citing a preference for THC or CBD, or citing no preference for either.

**Table 5:** Adjusted odds of remaining in the study at M6 by variables found to be significant in univariate regression analysis

Significant variables	Multivariate regression analysis		
	**AOR**^**a**^	**95% CI**^**b**^	***P***
**Use of opioid**			
N	—	—	
Y	0.62	0.46, 0.85	0.003
**Use of antidepressant**			
N	—	—	
Y	1.42	0.98, 2.07	0.061
**Use of anti-seizure**			
N	—	—	
Y	1.91	1.30, 2.81	< 0.001
**WHOQOL—psychological (baseline)**			
< 54	—	—	
≥ 54	1.43	1.08, 1.90	0.013
**Preferred type of cannabis**			
CBD	—	—	
THC	0.87	0.59, 1.27	0.5
No preference	1.16	0.82, 1.63	0.4
**Primary method of use**			
Inhaled	—	—	
Orally ingested	0.87	0.64, 1.19	0.4
**Enrollment period**			
Enrolled prior to 17 April 2018 (M6 would be pre-legalization)	—	—	
Enrolled between 17 April and 15 July 2018 (M6 would be post-legalization)	0.28	0.18, 0.41	< 0.001

^a^*AOR*  Adjusted odds ratio, ^b^*CI* Confidence interval

## Discussion

In this study, we found that non-medical cannabis legalization was independently associated with retention in the Tilray Observational Patient Survey. Additionally, the survival analysis and subsequent logistic regressions identified specific participant characteristics that were associated with the percentage, probability, and adjusted odds of retention at M6. Specifically, the use of opioids was strongly associated with greater probability and adjusted odds that participants would be LTFU before completing the study, while a higher baseline psychological score and the use of anti-seizure medications and were both associated with increased retention at M6.

The finding that the adjusted odds of participants being LTFU were three-fold higher compared to the pre-legalization period is consistent with other data examining the impacts of legalization on medical cannabis access in Canada. National polling data from January 2019 that included over 800 adult Canadian medical cannabis patients found that 26% of patients reported that medical cannabis had become more difficult to access since legalization, and that 48% of patients polled accessed cannabis via the illegal market, while 44% accessed cannabis via the legal non-medical market (Abacus 2019). Furthermore, a study of Canadian cancer patients that compared medical cannabis access and use both before (*n* = 821) and after legalization (*n* = 852) cited significant challenges obtaining cannabis products post-legalization (Hawley 2020). News reports from that time and subsequent academic studies suggest that industry-wide shortages of both legal medical and non-medical cannabis products likely resulted from the emergence of novel licensed retail outlets and online sales channels and the associated increase in demand, which diluted the available legal supply to both medical and non-medical markets channels (Armstrong 2019a, b, 2021). The lack of authorized cannabis products in the months immediately following legalization led Ontario, Canada’s most populous province, to temporarily halt the licensing of legal storefront dispensaries (Armstrong 2019b; Mazur 2019). News reports suggest this shortage lasted for many months, and likely led to a diversion of medical and non-medical cannabis consumers to access via illicit channels (Armstrong 2019a). While there were no measures in this study assessing whether patients LTFU in TOPS left the care of the participating clinics, stopped using medical cannabis, or simply accessed it from alternative sources, it is reasonable to presume that policy changes having a national impact on legal access to medical cannabis in Canada would also affect authorized patients involved in prospective medical cannabis studies over the same period, and that the reported product shortages provide a rationale for the significant decrease in retention seen in TOPS post-legalization.

While the supply situation appears to have been rectified, data from Health Canada highlights an 18% decline in patients registered in the federal medical cannabis program between September 2019 and June 2020 (the latest available data at the time of writing), suggesting the patient migration away from the federal medical cannabis program may be a longer-term trend (Health Canada 2020). That same period saw a steady expansion of regulated community-based non-medical retail outlets offering a large selection of different products at prices that are often lower than those in the legal medical market, which stands in sharp contrast to the online-only medical cannabis market that many patients have long complained is too expensive and difficult to access (Belle-Isle et al. 2014; Capler et al. 2017; Valleriani et al. 2020). However, there are some policy options the federal government could consider that might address some of the perceived shortcomings of the federal medical cannabis program, including allowing pharmacy-based access, which would have the important ancillary benefit of increased engagement with health care providers, since pharmacists could provide in-person information about safe use, adverse events, and potential contraindications. Additionally, reducing the cost of medical cannabis by extending the tax-exempt status of other prescription medicines in Canada to all medical cannabis products, and/or by expanding opportunities for cost coverage via private or public payers could also incentivize patients to access cannabis products via the medical system, as has been the case in Germany (Pascual 2020), thereby reducing the odds that patients enrolled in prospective medical cannabis studies would opt out of the federal medical cannabis program and subsequently be LTFU.

The outcomes of this study also suggest that patient capacity and study burden may have impacted retention. While only a few variables were shown to be associated with adjusted odds of retention at M6, most seem to be consistent with characteristics that may be indicative of overall patient physical/psychological capacity. Primarily, the finding that baseline opioid use reduced survival/retention in the TOPS study could be reflective of a patient population with less stable physical and psychological health conditions sometimes associated with or resulting from chronic opioid therapy (Baldini et al. 2012; Dobscha et al. 2013; Sullivan 2018), and which research has shown may impact retention in prospective studies (Zweben et al. 2009; O’Connor et al. 2020). Similarly, the finding that having a higher baseline score for the psychological measure of WHOQOL-Bref was associated with increased odds of retention further suggests that patient psychological capacity may have been a significant internal factor affecting the odds of patients being LTFU in TOPS. The only relevant finding that appeared unrelated to patient physical or psychological capacity was that the use of anti-seizure medications was associated with greater odds of retention. This outcome may be reflective of the well-established relationship between treatment adherence, reduced seizure frequency and severity, and improved quality of life in patients affected by seizure disorder (Sancho et al. 2010; Lin et al. 2016; Hamedi-Shahraki et al. 2019), which may have increased the motivation of this particular patient cohort to continue using cannabis within the scope of the federal medical cannabis program, and to subsequently remain in the study as well.

While recruiting participants with greater capacity could improve retention in future medical cannabis studies, it might also lead to recruitment bias and ultimately confound findings. Additionally, since such a significant portion of medical cannabis patients also use opioids (Campbell et al. 2018; Chen et al. 2019; Lucas et al. 2019; Safakish et al. 2020), it would be hard to justify excluding these patients from future research. Therefore, a better option to improve retention would be find ways to reduce study burden on this vulnerable patient population. Studies and systematic reviews specifically examining retention in prospective studies have found that study burden is one of the most significant factors affecting the odds of patients being LTFU, and have identified a number of barrier-reduction strategies that can significantly increase retention (Kearney et al. 2017; Sommer et al. 2018; Teague et al. 2018; Naidoo et al. 2020). These include reducing the number of study visits and associated assessments, improving patient compensation, and providing flexibility in data collection methods such as conducting follow-ups via web-based surveys, phone, or telemedicine (Zweben et al. 2009; Abshire et al. 2017; Svendsen et al. 2017; Teague et al. 2018).

This study has a number of strengths and limitations. This was a convenience sample recruited at 21 medical clinics across 5 Canadian provinces, and although the primary patient characteristics are consistent with similar studies of medical cannabis patient populations in Canada, Europe, Australia, and the USA (Hazekamp et al. 2013; Baron et al. 2018; Boehnke et al. 2019; Lintzeris et al. 2020), there is no guarantee this sample is representative of the general Canadian medical cannabis patient population, thereby limiting the generalizability of these findings. Since many of the clinics specialize in the treatment of chronic pain, there may be an over-representation of patients affected by chronic pain. Data regarding the use of cannabis was self-reported by patients and did not benefit from biological drug detection to confirm use or non-use of cannabis, so is subject to potential recall bias.

These limitations are counterbalanced by several methodological strengths, including the large number of participants (to the best of our knowledge this is the largest national prospective survey of Canadian medical cannabis patients to date), gathering of highly detailed data on patterns of cannabis and prescription drug use, and data entry of prescription drug use by physicians, rather than relying on patient self-report.

## Conclusions

Cannabis legalization in Canada has successfully shifted the regulation of cannabis from a predominantly criminal justice approach to one focused on public health and harm reduction. However, an ancillary and perhaps unexpected outcome has been its subsequent impact on patient access to medical cannabis, and associated efforts to study this population. While this analysis cannot determine if the association between legalization and reduced odds of retention in TOPS is unique to this study and/or the period immediately following legalization or indicative of a broader trend, researchers conducting prospective studies on cannabis in Canada and around the globe should anticipate and attempt to mitigate the potential impacts of increased access options on study retention. Evidence-based barrier reduction strategies that could reduce the odds of patients being lost to follow-up include minimizing the number of study visits and assessments, ensuring adequate patient compensation, and conducting follow-ups via online surveys, phone-based interviews, and telemedicine. Should increased access options continue to erode patient participation in Canada’s federal medical cannabis program and subsequently in studies assessing the harms and benefits of medical cannabis, pragmatic policy solutions designed to better meet the needs of patients—such as pharmacy-based access and increased cost-coverage—could form part of a more comprehensive strategy to improve patient access to medical cannabis and improve retention in prospective studies of this population.

## Supplementary Information

**Additional file 1.** TOPS Health Care Provider Talking Points.

## Data Availability

The datasets used and/or analyzed during the current study are available from the corresponding author on reasonable request.

## Abbreviations

AOR: Adjusted odds ratio
CBD: Cannabidiol
CI: Confidence interval
CUS: Cannabis Use Survey
DDD: Defined daily dose
LTFU: Lost to follow-up
M1: Month 1 data point
M3: Month 3 data point
M6: Month 6 data point
MME: Morphine milligram equivalent
NDDF: National Drug Data File
OR: Odds ratio
*p*: *p* value
PDQ: Prescription Drug Questionnaire
QOL: Quality of life
THC: Tetrahydrocannabinol
TOPS: Tilray Observational Patient Study
VAS: Visual assessment scale
WHOQOL-Bref: World Health Organization Quality of Life Short Form
