# Supplementary material for: The impact of non-medical cannabis legalization and other exposures on retention in longitudinal cannabis research: a survival analysis of a prospective study of Canadian medical cannabis patients
Source: J Cannabis Res. 2021 Jul 28;3:34. doi: 10.1186/s42238-021-00089-7 (PMC8320176; doi:10.1186/s42238-021-00089-7)
Supplement: Supplementary file 1 — Additional file 1. TOPS Health Care Provider Talking Points. [file 42238_2021_89_MOESM1_ESM.docx]

**Health Care Provider Talking Points**

Once a patient and physician agree that medical cannabis is an appropriate treatment option for the patient, the physician can present the TOPS project to the patient by using the following talking points:

- TOPS is a study being conducted by Tilray, a federally authorized Licensed Producer of medical cannabis.
- The study is an online survey protocol to examine the impact of medical cannabis on quality of life and prescription drug use.
- While we are assisting with the recruitment and administration of the TOPS study, this project is sponsored and conducted by Tilray.
- Your services and relationship with either me/this clinic or Tilray will not be affected in any way should you chose not to participate in this study, and/or to withdraw at a later point in time.

For in-person visits:

- If you’re interested in learning more about the TOPS study, I’d like to provide you with an informed consent form on an iPad that will provide more information about the study, and provide you with an opportunity to participate if you chose.

For telemedicine:

- If you’re interested in learning more about the TOPS study, I’d like to read you an informed consent form that will provide more details about the study, and provide you with an opportunity to participate if you chose.
